# Supplementary material for: Human and bacterial genetic variation shape oral microbiomes and health
Source: Nature. 2026 Jan 28;651(8105):429–39. doi: 10.1038/s41586-025-10037-7 (PMC12979206; doi:10.1038/s41586-025-10037-7)
Supplement: Supplementary file 2 — Reporting Summary [file 41586_2025_10037_MOESM2_ESM.pdf]

nature portfolio

Corresponding author(s): Nolan Kamitaki

Last updated by author(s): Nov 24, 2025

Reporting Summary

Nature Portfolio wishes to improve the reproducibility of the work that we publish. This form provides structure for consistency and transparency in reporting. For further information on Nature Portfolio policies, see our Editorial Policies and the Editorial Policy Checklist.

Statistics

For all statistical analyses, confirm that the following items are present in the figure legend, table legend, main text, or Methods section.

n/a	Confirmed	
☐	☒	The exact sample size (*n*) for each experimental group/condition, given as a discrete number and unit of measurement
☐	☒	A statement on whether measurements were taken from distinct samples or whether the same sample was measured repeatedly
☐	☒	The statistical test(s) used AND whether they are one- or two-sided<br>*Only common tests should be described solely by name; describe more complex techniques in the Methods section.*
☐	☒	A description of all covariates tested
☐	☒	A description of any assumptions or corrections, such as tests of normality and adjustment for multiple comparisons
☐	☒	A full description of the statistical parameters including central tendency (e.g. means) or other basic estimates (e.g. regression coefficient) AND variation (e.g. standard deviation) or associated estimates of uncertainty (e.g. confidence intervals)
☐	☒	For null hypothesis testing, the test statistic (e.g. *F*, *t*, *r*) with confidence intervals, effect sizes, degrees of freedom and *P* value noted<br>*Give P values as exact values whenever suitable.*
☒	☐	For Bayesian analysis, information on the choice of priors and Markov chain Monte Carlo settings
☒	☐	For hierarchical and complex designs, identification of the appropriate level for tests and full reporting of outcomes
☐	☒	Estimates of effect sizes (e.g. Cohen's *d*, Pearson's *r*), indicating how they were calculated

Our web collection on statistics for biologists contains articles on many of the points above.

Software and code

Policy information about availability of computer code

Data collection	CFX Manager (v.3.1) as associated with CFX384 Real-Time PCR Detection System was used to collect fluorescence measurements for amylase enzyme activity.
Data analysis	The following publicly available software resources were used: MetaPhlAn (v.4.0.6, http://segatalab.cibio.unitn.it/tools/metaphlan/index.html), DeepVariant (v.1.3.0, https://github.com/google/deepvariant), GLnexus (v.1.4.1, https://github.com/dnanexus-rnd/GLnexus), HUMAnN (v.3.8, https://huttenhower.sph.harvard.edu/humann), GraPhlAn (v.1.1.3, http://segatalab.cibio.unitn.it/tools/graphlan/index.html), mosdepth (v.0.3.6, https://github.com/brentp/mosdepth), bowtie (v.2.5.1, https://bowtie-bio.sourceforge.net/bowtie2/index.shtml), bcftools (v.1.14, http://www.htslib.org/), samtools (v.1.15.1, http://www.htslib.org/), plink (v.1.90b6.26 and v.2.00a3.7,https://www.cog-genomics.org/plink/), BOLT-LMM (v.2.4.1, https://alkesgroup.broadinstitute.org/BOLT-LMM/), qqman (v.0.1.8, https://cran.r-project.org/web/packages/qqman/index.html), MDMR (v.0.5.2, https://cran.r-project.org/web/packages/MDMR/index.html), bedtools (v.2.27.1, https://bed-tools.readthedocs.io/en/latest/), AlphaFold3 (v3, https://alphafoldserver.com/), and ChimeraX (v.1.9, https://www.cgl.ucsf.edu/chimerax/). Custom code used to generate results in this study is available via Zenodo at 10.5281/zenodo.14559458

For manuscripts utilizing custom algorithms or software that are central to the research but not yet described in published literature, software must be made available to editors and reviewers. We strongly encourage code deposition in a community repository (e.g. GitHub). See the Nature Portfolio guidelines for submitting code & software for further information.

Data

Policy information about availability of data

All manuscripts must include a data availability statement. This statement should provide the following information, where applicable:

- Accession codes, unique identifiers, or web links for publicly available datasets
- A description of any restrictions on data availability
- For clinical datasets or third party data, please ensure that the statement adheres to our policy

The following data resources are available by application: UKB (http://www.ukbiobank.ac.uk/), All of Us Research Program (https://allofus.nih.gov/), and SFARI SPARK (https://www.sfari.org/resource/spark/). Relative abundances of species in the oral microbiome will be returned to SFARI for release upon request. The following data resources are publicly available: Human Microbiome Project (https://hmpdacc.org/), human reference genome build GRCh38 (https://ftp.1000genomes.ebi.ac.uk/vol1/ftp/technical/reference/GRCh38_reference_genome/), MetaPhlAn vOct22 reference database (http://cmprod1.cibio.unitn.it/biobakery4/metaphlan_databases/), TOPMed-r3 imputation panel (https://imputation.biodatacatalyst.nhlbi.nih.gov/), LD score resources (https://alkesgroup.broadinstitute.org/LDSCORE/), NCBI GenBank (https://www.ncbi.nlm.nih.gov/genbank/), and NCBI Conserved Domain Database (https://www.ncbi.nlm.nih.gov/Structure/cdd/cdd.shtml).

Research involving human participants, their data, or biological material

Policy information about studies with human participants or human data. See also policy information about sex, gender (identity/presentation), and sexual orientation and race, ethnicity and racism.

Reporting on sex and gender	Sex was used as a covariate in several analyses, but no values directly pertaining to sex are reported.
Reporting on race, ethnicity, or other socially relevant groupings	For UK Biobank, using the top 20 ancestry principal components, a subset of individuals that fell within a Euclidean distance (centered at the mean values of each PC for individuals who self-identified as "white") capturing 99% of individuals who self-identified as "white" were used for phenotype associations. For All of Us, analyses were performed either on the entire cohort, or by restricting to released genetically-predicted ancestry as noted. For SPARK, all individuals were included in analyses without restricting to any ancestry. For all analyses except associations with microbial gene dosage, ancestry principal components were included as covariates in genetic associations.
Population characteristics	UK Biobank is a cohort of approximately 500,000 individuals across the United Kingdom between 40 and 69 years of age at time of recruitment (Sudlow et al. 2015 PLOS Medicine). For phenotype associations in the UK Biobank cohort, age, age squared, sex, genotype array, assessment center, and top 20 genetic ancestry PCs were used as covariates. All of Us is a cohort of approximately 245,000 individuals with WGS available (at time of analysis) across the United States older than 18 years of age at time of recruitment (The All of Us Research Program Investigators 2019 N Engl J Med). For oral health associations in the All of Us cohort, age, age squared, sex, and the top 16 genetic ancestry principal components (from ancestry_preds.tsv) were used as covariates. For BMI associations in the All of Us cohort, only genetic ancestry principal components were used as covariates as age and sex had already been residualized out. SFARI SPARK is a cohort of approximately 160,000 families with at least one child with autism spectrum disorder, where 12,519 individuals (at time of analysis) have WGS from saliva available (SPARK Consortium 2018 Neuron). For microbiome associations in the SPARK SFARI cohort, sequencing batch, age, age squared, square root of age, sex, percent of mapped reads, and the top 10 genetic ancestry principal components were used as covariates.
Recruitment	Individuals and biosamples were not obtained for this study and their recruitment is as described in prior publications (cited in current work).
Ethics oversight	Individuals and biosamples were not obtained for this study and local IRBs at each institution approved the collections and patient-consent materials, as described in the earlier papers on these cohorts (cited in current work). North West-Haydock Research Ethics Committee gave ethical approval for UK Biobank data collection and availability under reference 16/NW/0274. Western IRB of Wayne State University gave ethical approval for Simons Foundation Autism Research Initiative (SPARK) data collection and availability under protocol 20151664. The IRB of the All of Us Research Program gave ethical approval gave ethical approval for All of Us data collection and availability under protocol 2021-02-TN-001. The Office of Research Subject Protection (ORSP) of the Broad Institute waived ethical approval for this work, as this research on de-identified, previously-collected data was determined not to constitute human subjects research and did not require IRB review. Data from the UKB Resource were accessed under application number 40709 and from SFARI SPARK under application 3350.2.

Note that full information on the approval of the study protocol must also be provided in the manuscript.

Field-specific reporting

Please select the one below that is the best fit for your research. If you are not sure, read the appropriate sections before making your selection.

☒ Life sciences ☐ Behavioural & social sciences ☐ Ecological, evolutionary & environmental sciences

For a reference copy of the document with all sections, see nature.com/documents/nr-reporting-summary-flat.pdf

Life sciences study design

All studies must disclose on these points even when the disclosure is negative.

Sample size	Starting from 488,377 individuals in the UK Biobank SNP-array data set, individuals were excluded based on the following criteria: 36,008 were removed to drop one relative within pairs of close relatives with kinship coefficient > 0.0884, preferentially keeping individuals if they a) reported having dentures or b) reported not having dentures (i.e., had a non-missing dentures phenotype); 28,701 were removed for not having European genetic ancestry; 1,469 were removed for not having available TOPMed-imputed genotypes (including for chromosome X); 2,601 were removed for not having available WGS data; and 53 were removed for having withdrawn, leaving 419,545 available individuals for genetic association analyses. For the binary oral health phenotypes (dentures use and bleeding gums), 418,039 had non-missing values. For the quantitative BMI z-score phenotype, 418,150 had non-missing values.<br><br>For the All of Us cohort, 245,377 samples were genotyped for AMY1 copy number from available WGS data and these were then filtered to an unrelated subset of samples (iteratively dropping one individual per related pair with kinship score > 0.1, from relatedness_flagged_samples.tsv). 230,002 individuals had non-missing values for the oral health phenotypes and 219,879 individuals had non-missing values for the BMI z-score phenotype. For replication of microbial gene dosage associations with human genetic variants a random set of 10,000 samples with saliva as the biosample type were chosen.<br><br>For the SFARI SPARK cohort, all 12,519 samples with available WGS data were used for all analyses unless otherwise specified (ex. subsets used with sufficient genomic read depth coverage of a specific microbial species).<br><br>In all cases except for replication of microbial gene dosage associations in All of Us, no sample-size calculation was done to predetermine sample size and the maximum number of available samples were used. For oral health associations (UK Biobank), we expected that the association would be sufficiently powered to allow for associating AMY1 copy number with dentures risk given nearby variants reached genome-wide significance and low r2 (<0.2) between AMY1 copy number and any biallelic tag variants. Additionally, we expected reasonable power to find evidence of colocalized dentures use associations with microbiome composition given the large number of genome-wide significant loci (n=47) seen in a previous GWAS for dentures risk. For BMI associations, we expected reasonable power to replicate previously reported associations with AMY1 copy number, given each of our cohorts (UK Biobank and All of Us) were nearly two orders of magnitude larger than the largest where a significant relationship was observed. For oral microbiome associations, we expected comparable power to find significantly associated human loci given results from several similarly sized gut microbiome association studies (n=8-16k). For replication of microbial gene dosage associations in All of Us, we chose a sample size of 10,000 to limit computational expense while approximating power of the SFARI SPARK cohort. For enzymatic assays of amylase isoforms, we expected 32 replicates to be sufficient to observe an effect sufficient to explain the genotypic associations (22.4- and 7.3-fold).
Data exclusions	Established QC metrics were used to exclude some samples, genotypes, or sequencing data for analysis as described in previously published studies (cited in the current work). Samples from individuals in UK Biobank, All of Us, and SFARI SPARK that requested to be withdrawn at the time of analysis were excluded.
Replication	For oral health phenotypes, All of Us (complete tooth loss, caries) served as independent replicate for association with AMY1 copy number as first performed in UK Biobank (dentures use), where caries was previously reported to have high genetic correlation with dentures use. Additionally, although not genome-wide significant, the bleeding gums phenotype in UK Biobank also served as a replication of the AMY1 copy number allelic series with effects from missense variants (F141C, C477R).<br><br>For BMI z-score phenotype, All of Us served as an independent replicate for association with AMY1 copy number as first performed in UK Biobank. Additionally, the same lack of association with AMY1 copy number seen in each genetically-predicted ancestry of All of Us serve as confirmations of non-ancestry specific trends.<br><br>For microbiome composition associations, the colocalization of individually significant microbial species at the same human genetic loci (even at a (taxa)x(human genetic variants) level of Bonferroni correction) each replicate the overall pattern of association, but also often resolved to the same lead index variant and pattern of association (Fig. 2, 3 and Extended Data Fig. 2, 3). Additionally, the comparison of associations for species relative abundance in adults and children separately (performed to assess the plausibility of reverse causality from dentures use) serve as additional replicates of the human genetic effect. The relative effect sizes of F141C and C477R on the relative abundance of different microbial species also affected by AMY1 copy number (Fig. 3i) further replicates these variants as exerting some phenotypic function equivalent to additional copies of AMY1.<br><br>For microbial gene dosage associations, the effects observed in the SFARI SPARK cohort were replicated in an independent set of 10,000 samples from the All of Us cohort (Extended Data Fig. 9c)<br><br>For in vitro amylase enzymatic assays, purified protein from reference sequence and F141C AMY1 isoforms was used in n=32 replicates of enzymatic activity, where all attempts were successful and included in Extended Data Figure 7c.
Randomization	For UK Biobank, samples were collected in batches at different assessment centers at locations across the United Kingdom and these were encoded as indicator covariates in phenotype-genotype associations. For SFARI SPARK, samples were collected in sequencing batches (WGS1 through WGS5), where these were encoded as indicator covariates. For All of Us, samples were sequenced in batches at different centers, where these were encoded as indicator covariates. For enzymatic assays, replicates were run as prepared in equally sized groups on plates, where plates were encoded as indicator covariates. No further randomization was done as all samples were used for each analysis.
Blinding	For all computational analyses, samples were listed with a randomized ID where association of measured genotype with trait (phenotype such as dentures use or relative abundance of a particular microbial species) was only done at the point of final statistical analysis. Blinding was not done for in vitro amylase enzymatic assay sample plating, as fluorescence quantification was performed simultaneously and identically for all samples on each plate.

Reporting for specific materials, systems and methods

We require information from authors about some types of materials, experimental systems and methods used in many studies. Here, indicate whether each material, system or method listed is relevant to your study. If you are not sure if a list item applies to your research, read the appropriate section before selecting a response.

Materials & experimental systems

n/a	Involved in the study
☐	☒ Antibodies
☐	☒ Eukaryotic cell lines
☒	☐ Palaeontology and archaeology
☒	☐ Animals and other organisms
☒	☐ Clinical data
☒	☐ Dual use research of concern
☒	☐ Plants

Methods

n/a	Involved in the study
☒	☐ ChIP-seq
☒	☐ Flow cytometry
☒	☐ MRI-based neuroimaging

Antibodies

Antibodies used: anti-Amylase Antibody (clone G-10, Santa Cruz Biotechnology, catalog no. sc-46657, lot no. G0324), Anti-mouse IgG, HRP-linked Antibody (Cell Signaling Technology, catalog no. 7076, lot no. 39)

Validation: The anti-Amylase Antibody (clone G-10) has been validated for use in Western blotting against human salivary amylase with some user-submitted Western blots (ex. Luti, S. et al. Chronic Training Induces Metabolic and Proteomic Response in Male and Female Basketball Players: Salivary Modifications during In-Season Training Programs. Healthcare (Basel) 11, 241 (2023).). We also confirm a) it recognizes a single band at the expected size (~56 kD) in glycogen-purified cell culture supernatant (EDF 6) which is expected to yield only amylase from previous work, and b) presence of this band in unpurified cell culture supernatant from cells transfected with a plasmid containing amylase coding sequence but not those without. We do note that there is a non-specific band at ~50kD present in the supernatant of cells transfected with a control plasmid not containing AMY1. The anti-mouse antibody (CST 7076) has been validated against CST primary antibodies in Western blots as indicated on the manufacturer's website.

Eukaryotic cell lines

Policy information about cell lines and Sex and Gender in Research

Cell line source(s): Lenti-X 293T (HEK293T clone) from Takara Bio USA (lot no. AIY00015, catalog no. 632180)

Authentication: Morphological match for type and in-house verification of SV40T antigen with genotyping PCR assay. No other standard authentication methods were performed (such as STR typing).

Mycoplasma contamination: Lack of mycoplasma contamination was done by Takara Bio USA as well as by members of receiving lab (McCarroll)

Commonly misidentified lines (See ICLAC register): None were used in this study, HEK293T is a derivative of HEK and has not been listed as commonly misidentified.

Plants

Seed stocks: *Report on the source of all seed stocks or other plant material used. If applicable, state the seed stock centre and catalogue number. If plant specimens were collected from the field, describe the collection location, date and sampling procedures.*

Novel plant genotypes: *Describe the methods by which all novel plant genotypes were produced. This includes those generated by transgenic approaches, gene editing, chemical/radiation-based mutagenesis and hybridization. For transgenic lines, describe the transformation method, the number of independent lines analyzed and the generation upon which experiments were performed. For gene-edited lines, describe the editor used, the endogenous sequence targeted for editing, the targeting guide RNA sequence (if applicable) and how the editor was applied.*

Authentication: *Describe any authentication procedures for each seed stock used or novel genotype generated. Describe any experiments used to assess the effect of a mutation and, where applicable, how potential secondary effects (e.g. second site T-DNA insertions, mosiacism, off-target gene editing) were examined.*
